# Large Water Management Projects and Schistosomiasis Control, Dongting Lake Region, China

**DOI:** 10.3201/eid1307.070848

**Published:** 2007-07

**Authors:** Yue-Sheng Li, Giovanna Raso, Zheng-Yuan Zhao, Yong-Kang He, Magda K. Ellis, Donald P. McManus

**Affiliations:** * Hunan Institute of Parasitic Diseases, Yueyang, People’s Republic of China; † Queensland Institute of Medical Research, Brisbane, Queensland, Australia; ‡ University of Queensland, Brisbane, Queensland, Australia; § Central South University, Changsha, People’s Republic of China

**Keywords:** China, Dongting Lake, Hunan Province, Oncomelania, parasite control, “Return Land to Lake” Program, schistosomiasis, Three Gorges Dam, water management, synopsis

## Abstract

Construction of the Three Gorges Dam across the Yangtze River will substantially change the ecology of the Dongting Lake in southern China. In addition, the Central and Hunan Provinces’ governmental authorities have instigated a Return Land to Lake Program that will extend the Dongting Lake surface area from the current 2,681 km^2^ to 4,350 km^2^.The previous construction of embankments and the large silt deposits made by the Yangtze River and other connecting rivers have contributed to frequent disastrous flooding. As a consequence of the 2 water projects, >2 million persons and their domestic animals are being resettled. This article provides an overview of the historical background of these 2 large water management projects, the associated population movement, and their impact on future transmission and control of schistosomiasis in the Dongting Lake area. The dam will likely substantially extend the range of the snail habitats and increase schistosome transmission and schistosomiasis cases.

## Abstract 2

Two large water projects will likely extend the range of snail habitats and increase schistosome transmission.

Over a period of 2,200 years, until 1911, the Yangtze River has flooded 214 times, an average of 1 flood every 10 years. In the past century, 5 such floods have been severe. With the development of the Yangtze Basin, the economic cost of such flooding has also increased substantially. Controlling the flow of the Yangtze through the Three Gorges area would substantially reduce the danger of flooding in the lower plains regions and thus ameliorate economic losses. The construction of the giant Three Gorges Dam was first proposed in 1919, but construction did not begin until 1994. The entire Three Gorges project will be completed in 2009, and the dam’s generated power is expected to supply ≈10% of the electricity needs of the People’s Republic of China. After completion, the dam will be 2,300 m long and 185 m high, and the resulting 600-km-long reservoir created by the dam will inundate 115,000 acres of cultivated land. Hundreds of villages and countless historical relics and archeological and cultural sites will be submerged; ≈1.4 million persons will be resettled from this area (*1*–*3*). The construction of the dam will change the distribution of water and sand downstream from the dam and thus will have a strong effect on ecologic systems such as Dongting Lake in Hunan Province (Figure 1).

**Figure 1:**
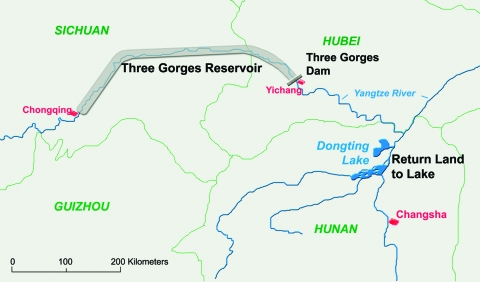
Location of the Three Gorges Dam and Reservoir across the Yangtze River and Return Land to Lake Program in the Dongting Lake region, Hunan Province, China.

Dongting Lake is located at 28°30′–30°20′ N and 111°40′–113°40′ E in the northeastern part of Hunan Province and covers a water surface area of 2,681 km^2^ (*3*). The heavily populated Dongting Lake basin is one of China’s leading rice-producing regions; it is also known for its production of cotton and fish. Dongting Lake was China’s largest lake during the Han dynasty. The rich sediment of the marshland attracted farmers, and several embankments were built to keep out the Yangtze River and to gain more farmland. Unfortunately, silting of mud and sand in the lake, in addition to the anthropogenic environmental transformations in the lowland areas, reduced the lake area and its storage capacity and caused rapid deterioration of the lake’s flood diversion and flood storage functions. This diminishing capacity increased the occurrence of flood disasters, mainly because of the rupture of embankments. Until 1998, the lake had 228 embankments and was surrounded by a farmland area of 0.34 million ha. After a disastrous flood in 1998 (Figure 2A, B), which led to 3,656 deaths, made 378,000 persons homeless, and resulted in an economic loss of US $737 million, the State Council of the People’s Republic of China formulated a policy, the Return Land to Lake Program, to prevent flooding. This program envisioned returning cultured lands into lake areas and included removing embankments and relocating residents from schistosomiasis-endemic areas to newly established towns. As a direct result, the Hunan government initiated a huge 4-year environmental project. The project involves moving 815,000 inhabitants of the Dongting Lake area inland and will result in the loss of >900,000 ha of farmland. Today, almost 90% of this project is completed (*4*,*5*).

**Figure 2:**
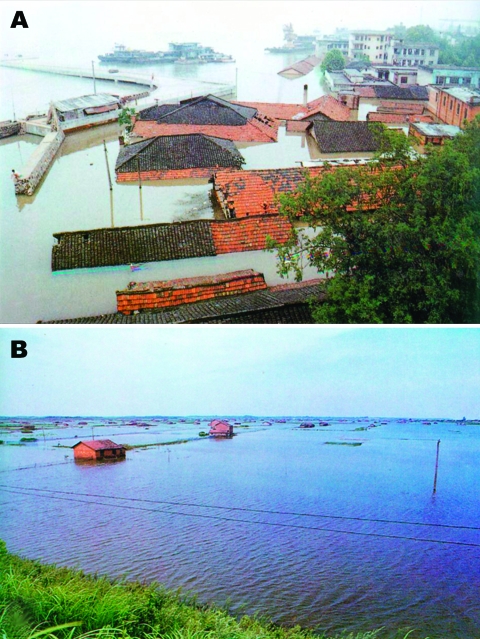
A) Submerged township houses in the Dongting Lake area due to the flood of 1998. B) An inundated rural area in 1998.

As a consequence of these 2 huge water management projects, >2 million people and their domestic animals are being resettled. Large population movements are associated with extreme vulnerability to disease, especially due to malnutrition and lack of access to clean water and appropriate sanitation. Furthermore, officials are concerned that these water management projects will increase the transmission of schistosomiasis (*2*,*6*,*7*).

## Schistosomiasis in Dongting Lake Area

Schistosomiasis japonica is a zoonotic parasitic disease caused by the trematode blood fluke *Schistosoma japonicum*, whose life cycle includes an amphibious freshwater snail, *Oncomelania hupensis hupensis*. The infection is transmitted by cercariae released by the snails and is contracted percutaneously by humans and other mammalian hosts, notably water buffaloes, when they are exposed to infested water. Schistosomiasis is a serious disease, which may become chronic, and remains a major health risk for the estimated >50 million persons living in the tropical and subtropical zones of China (7).

**Figure 3:**
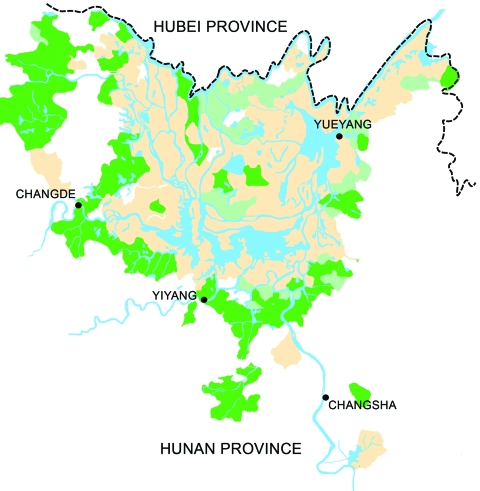
Hunan Province in 2002, showing that schistosomiasis is mainly confined to the area surrounding Dongting Lake. Areas in beige are classified as endemic for schistosomiasis. Areas in light green have fulfilled the transmission control criteria and are characterized by schistosomiasis infection rates <1% for humans and animals and a snail habitat reduction of >98%. Areas in dark green have fulfilled the criterion of interrupted transmission, which means that no new human or animal schistosomiasis cases occurred in successive years, and no snails were found for >1 year.

Archaeological studies have shown that schistosomiasis japonica has been endemic in the Dongting Lake region for thousands of years (*8*,*9*). In 1956, schistosomiasis was prevalent in 5 prefectures, 24 counties, and 14 state farms in the region; a total of 1 million persons and >300,000 domestic animals were infected with *S. japonicum,* and the total area of *Oncomelania* intermediate snail host habitats in the lake region was 3,795 km^2^. After nearly half a century of control efforts, schistosomiasis is still endemic in 34 counties, and 6.12 million people are at risk for infection around Dongting Lake (Figure 3). Nearly 20% buffaloes were infected, and 200,000 active human cases were reported in 2003 (*10*). In the Dongting Lake region, schistosomiasis japonica particularly affects certain occupational groups, notably, farmers and fishermen, thus having substantial effects on the local economy and agricultural development of the area (*11*).

The continued concerted efforts to control schistosomiasis in China have reduced the initial peak estimates of human prevalences by >90%, and the national schistosomiasis control program for China is recognized as one of the most successful worldwide (*6*,*12*,*13*). Despite these remarkable achievements, schistosomiasis remains endemic in major foci in the marsh and lake regions of southern China, which cover a vast area of 5 provinces (Jiangsu, Anhui, Hubei, Jiangxi, and Hunan) and 2 mountainous regions (Sichuan and Yunnan) (*7*). Current estimates suggest that 843,007 humans and 74,000 bovines are infected (*7*,*14*). High-risk areas occur particularly around the Dongting and Poyang Lakes in the middle and lower reaches of the Yangtze River. Infected persons within these areas account for 86% of the total number of persons infected in the whole of China (*15*). Recent data from Sichuan Province suggest that schistosomiasis is reemerging in areas where the disease had been eliminated (*16*). With regard to the Dongting and Poyang Lakes, human reinfection with *S. japonicum* remains unacceptably high. Annually, up to 14% of patients who receive treatment are likely to become reinfected (*11*,*17*).

## Water Management Projects and Schistosomiasis Transmission

As illustrated in a recent systematic review by Steinmann and colleagues, the development of water resources and their management can have increase schistosomiasis transmission (*18*). The meta-analysis, based on African studies, showed a risk ratio of 2.4 and 2.6 for urinary schistosomiasis (caused by *S. hematobium*) and intestinal schistosomiasis (caused by *S. mansoni*), respectively, among persons living adjacent to dam reservoirs. The analyses also showed that persons living near land that had been irrigated for agricultural use had an estimated risk ratio of 1.1 for urinary schistosomiasis and an estimated risk ratio of 4.7 for intestinal schistosomiasis. Furthermore, the same group estimated that 8.76 million Chinese persons live in irrigated, schistosome-endemic areas and that 9.97 million Chinese persons live in areas that are at high risk because of dam construction; however, they were unable to identify studies that assessed the effect of water resources development and management on schistosomiasis and called for additional screening of the Chinese literature (*18*).

To our knowledge, no published studies have yet actually measured the effect of the Three Gorges Dam on the transmission of schistosomiasis in the Dongting Lake region or in other water systems that connect with the 600-km-long reservoir resulting from the dam construction. However, several studies have used observed data on humans, animals, and snails in the region and knowledge gained from epidemiologic studies to predict the effect of ecologic changes on schistosomiasis transmission before and after construction of the dam. For example, sand and soil upstream of the dam are predicted to be deposited as silt in the Three Gorges reservoir (*17*,*19*), substantially reducing sand and soil downstream of the dam. Thus, one of the beneficial effects of the construction of the Three Gorges Dam would be an 85% reduction of yearly deposited sand and soil in Dongting Lake 50 years after closure**.** However, owing to less accumulation of silt deposits, a part of the marshland covered with reeds would degenerate into grass beaches or reed-grass beaches, which might increase snail breeding areas and potential for transmission of *S. japonicum*. In addition, the predictive models show that after 50 years, the sand and soil deposited in the Three Gorges reservoir would slowly discharge downstream, which might become a challenging problem for Dongting Lake in the future (*20*). Thus, the Three Gorges Dam will likely substantially extend the range of the snail habitats and increase schistosome transmission and the number of new schistosomiasis cases. Models inferred that when the dam is completed, the water level in Dongting Lake could increase by 0.06–1.5 m from January to May and decrease by 1.6–2.0 m from November to December. In the years soon after dam construction**,** the water level would not affect the distribution of snails. However, human and animal exposure to infested water can be expected to increase because the lake water would recede 3–7 days earlier than in previous years, and 17–25 days earlier than during drought periods (*20*). However, schistosomiasis surveillance and control cannot rely solely on such predictive models because the accuracy might be questionable; undertaking studies that measure the effect of the dam on *S. japonicum* transmission dynamics and schistosomiasis is thus of vital importance.

**Figure 4:**
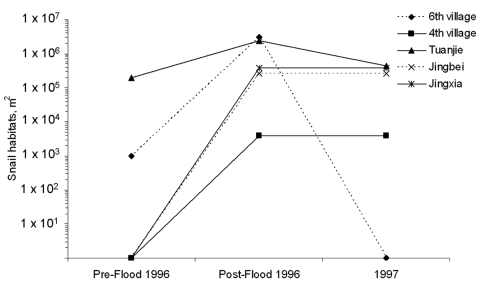
Influence of flooding in 1996 on *Oncomelania* snail distribution in 5 villages (data from [*21*]).

**Figure 5:**
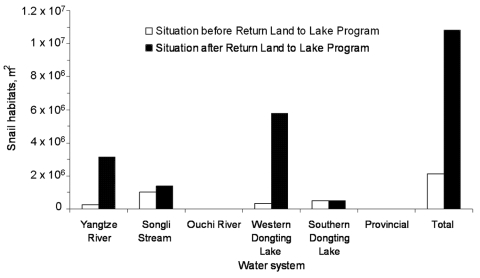
Influence of the Return Land to Lake Program on *Oncomelania* snail habitats in 6 different water systems in the Dongting Lake region (data from [*5*]).

In some areas where snails were eliminated through the construction of embankments, snail habitats have reappeared with the implementation of the Return Land to Lake Program flood prevention policy, which includes the removal of embankments. In fact, the lake embankments not only prevent flooding and increase land for cultivation but also minimize snail habitats inside the embankment. Preventing floods would automatically prevent *Oncomelania* snails from spreading throughout the lake region through breaking embankments. Such a spread of *Oncomelania* snails was documented in 5 villages when 1 year after the flood of 1996, antischistosomiasis workers visited a damaged embankment located in Huarong County, eastern Dongting Lake, and found that snails had spread to farmland areas (*21*) (Figure 4). The aforementioned concerns are confirmed by a study conducted in 41 villages distributed across different water systems of the Dongting Lake region (*5*). As shown in Figure 5, Cai and colleagues (*5*) showed that the Return Land to Lake Program had a negative effect on the *Oncomelania* snail distribution in 3 of the 6 investigated water systems. The authors estimated a >5-fold increase (from 0.2 km^2^ to 1.8 km^2^) of the snail areas due to the implementation of the program in the 6 investigated water systems of the Dongting Lake region (*5*). Overall, the area where the flood prevention policy is being implemented is as large as 6,670 km^2^ and it is estimated that snails will recolonize ≈90% of the area from which they were eliminated in the past. The current snail habitat areas will likely double in the coming 3–5 years in the lake regions of China (*22*). Notably, marsh restoration is also under way in southern Iraq (*23*), with potential for substantial resurgence in *S. hematobium* transmission.

**Figure 6:**
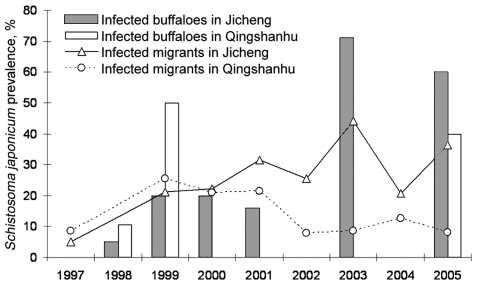
*Schistosoma japonicum* infection prevalences of migrants and water buffaloes in 2 areas in Dongting Lake over 9 years where the Return Land to Lake Program has been implemented (*27*). No bovine prevalence data were available for both villages for 1997, 2002, and 2004, and no human prevalence data were available for both villages for 1998. No buffaloes were present in Qingshanhu in 2000, 2001, and 2003.

**Figure 7:**
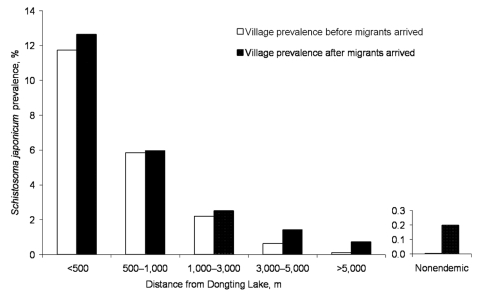
*Schistosoma japonicum* prevalences before and after migration due to the implementation of the Return Land to Lake Program in the Dongting Lake region.

As a direct result of the Return Land to Lake Program, thousands of farmers and fishermen and their domestic animals are being resettled inland from the Dongting Lake coastal areas. Most of these migrants will be engaged in daily activities such as agriculture, fishing, and reed cutting and will unavoidably have contact with water infested with schistosome cercariae. A recent drug-based intervention study showed that water buffaloes are the major host reservoir for transmission of *S. japonicum* to humans in the lake region of China and account for ≈80% of transmission; the highest numbers of eggs were released into the environment by young buffaloes <1 year of age (*24*). The use of domesticated buffaloes as working animals by farmers will continue to provide reservoirs for *S. japonicum*. Schistosome infections due to occupational exposure may therefore reemerge in areas where schistosomiasis was previously reduced or eliminated, while compliance for diagnostic testing and treatment in Dongting Lake communities can be low (*25*,*26*). A recent longitudinal study that looked at schistosomiasis prevalences over 9 years (1997–2006) among mobile populations in Jicheng and Qingshanhu villages in the Dongting Lake region confirms these concerns (Figure 6). Jicheng and Qingshanhu are located in areas where the Return Land to Lake Program has been implemented; these 2 locations were occupied over time by migrants who pursued their economic activities, including fishing and animal farming. As a result, the prevalence of schistosomiasis increased steadily among the migrants and their animals. In Jicheng, persons remained engaged in fishing and animal farming during the whole study period, whereas in Qingshanhu, persons switched to industrial fish cultivation after 2001. Fish cultivation is a protective factor; thus, schistosomiasis prevalence decreased among these migrants, whereas in Jicheng, the prevalence of the disease continued to increase. Another study (Y.-K. He, unpub. data) on Dongting Lake showed that after persons were relocated from disease-endemic areas to 27 disease-endemic or -nonendemic villages, disease prevalence substantially increased in the former (Figure 7). Schistosomiasis prevalence decreased as the distance between the villages and Dongting Lake increased. Furthermore, schistosomiasis was again endemic in villages where it had previously been successfully eliminated. Another study showed that people who had been relocated from the Dongting Lake to hilly areas were at a decreased risk for infection with *S. japonicum* (*28*). On the other hand, migrants from disease-nonendemic areas, or areas where schistosomiasis was controlled, who are relocated to endemic areas will be at a high risk for infection because of limited natural immunity (i.e., persons never exposed to schistosomiasis) or diminished immunity (i.e., persons formerly exposed). Official statistics indicate that 5 new rural communities have been built around Dongting Lake for >2,000 migrants relocated from the schistosomiasis-nonendemic areas upstream of the Three Gorges Dam area at Chongqing (Figure 1). These migrants will likely be at high risk for *S. japonicum* infection if they contact infested water.

## Future Control Strategies and Research Priorities

China’s rapid economic development has resulted in the decentralization and market orientation of the health system (*29**,**30*). Unfortunately, treatment and prevention of schistosomiasis, which was receiving policy and programmatic advantages such as the provision of free medication and prevention, will undoubtedly be affected in terms of both efficiency and equity (*29**,**30*). Although the Chinese population has some awareness of schistosomiasis and its health effects, a considerable proportion of persons have no knowledge of preventive measures or are unwilling to pay for treatment (*31*). The health system currently in place may not satisfactorily meet the distinct needs of the changing population in the Dongting Lake area; the situation may be further exacerbated by the large population movements that are currently taking place. Studies are therefore needed that assess and quantify demographic, environmental, and socioeconomic changes on health outcomes in connection with the relocation of the 2 large migrant populations associated with the Three Gorges Dam project and the Return Land to Lake Program. Findings from these studies would enable the Chinese authorities to estimate and locate the actual population at risk and thus help with the strategic planning of future control efforts. Furthermore, health education will have to play a leading role in the control of schistosomiasis, especially in those communities with little or no prior knowledge of prevention and control or of drug treatment and other control strategies. School- and community-based health education that builds on persons’ preexisting knowledge and perceptions has a strong potential for raising knowledge and awareness of schistosomiasis (*32**,**33*).

**Figure 8:**
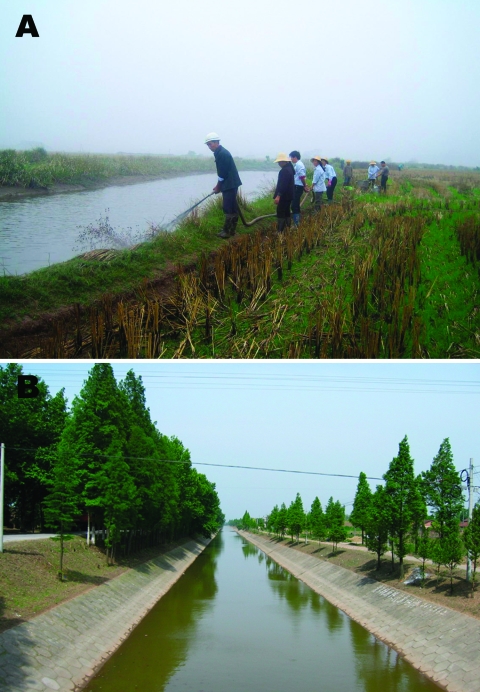
A) Mollusciciding with niclosamide for the control of *Oncomelania* snails in marshland between Dongting Lake and an embankment. B) Environmental modification to control *Oncomelania* breeding sites through canalization of water streams in Hunan Province.

In conclusion, integrated schistosomiasis control can be seen as a realistic target in the Dongting Lake region through strengthening of the schistosomiasis surveillance system already in place, which includes the monitoring of potential snail habitats, animal host reservoirs, and human cases. This monitoring is of central importance because with the implementation of the Return Land to Lake Program, local schistosomiasis endemicity might change (*4*). Current tools for controlling schistosomiasis may have to be combined or strengthened and strategies adapted according to social and environmental factors. Such strategies could include the following: improved access to treatment and preventive measures, health education, focal mollusciciding (Figure 8A), environmental modification (Figure 8B), and improvement of sanitation and water-supply systems through the efforts of intersectorial collaboration and local economic development.
